# Malrotated Subhepatic Caecum with Subhepatic Appendicitis: Diagnosis and Management

**DOI:** 10.1155/2016/6067374

**Published:** 2016-08-25

**Authors:** Hock Chin Chong, Feng Yih Chai, Dhayal Balakrishnan, Siti Mohd Desa Asilah, Irene Nur Ibrahim Adila, Khuzaimah Zahid Syibrah

**Affiliations:** ^1^Department of Surgery, Hospital Queen Elizabeth, Kota Kinabalu, Sabah, Malaysia; ^2^Department of Surgery, Hospital Keningau, Sabah, Malaysia; ^3^Department of Radiology, Hospital Keningau, Sabah, Malaysia

## Abstract

Subhepatically located caecum and appendix is a very rare entity. It occurs due to the anomaly in fetal gut rotation that results in an incomplete rotation and fixation of the intestine. Appendicitis, which is a common surgical emergency, in combination with the abnormal subhepatic location, presents a great challenge in its diagnosis and management. Here, we describe a 42-year-old male with chronic dyspepsia who presented with sepsis and severe pain at his right hypochondriac and epigastric region. The final diagnosis was acute appendicitis of the subhepatic appendix. Our discussion focuses on the diagnostic approach and clinical and surgical management. We hope that our report will increase the awareness among the clinicians and hasten the management of such rare condition to avoid complications.

## 1. Introduction

Acute appendicitis, which is a common surgical emergency, in combination with the rare subhepatic location, presents a significant challenge in its diagnosis and management. It can mimic cholecystitis and liver abscess, may lead to delayed diagnosis, and increases the chances of appendiceal rupture [1, 2].

Here, we describe a 42-year-old male with chronic dyspepsia who presented with sepsis and severe right hypochondriac and epigastric regions' pain. Our discussion focuses on the diagnostic approach and clinical and surgical management. We hope that our report will increase the awareness among clinicians of such a rare condition and hasten its management to avoid complications.

## 2. Case Presentation 

A 42-year-old male with chronic dyspepsia presented to us with acute severe epigastric and right hypochondriac region pain. He was afebrile with blood pressure of 117/78 mmHg and pulse rate of 103 beats/minute. On examination, his epigastrium and right hypochondriac region were tender, but there was no guarding. His haemoglobin was 15.8 g/dL, total white blood cells (TWBC) count was 16,300/*μ*L, and platelet was 254,000/*μ*L. His serum electrolytes, liver enzyme, and amylase levels were normal. Erect chest X-ray showed no air under the diaphragm. Subsequently, an abdominal ultrasound (US) was performed but only showed minimal free fluid at Morison's pouch with no other abnormality.

We suspected he had a sealed perforated peptic ulcer and proceeded to keep him nil by mouth, inserted a nasogastric tube to decompress his stomach, and started him on therapeutic intravenous cefuroxime and metronidazole with adequate analgesia and intravenous hydration while waiting for his computed tomography (CT) scan of abdomen and pelvis.

**Figure 1:**
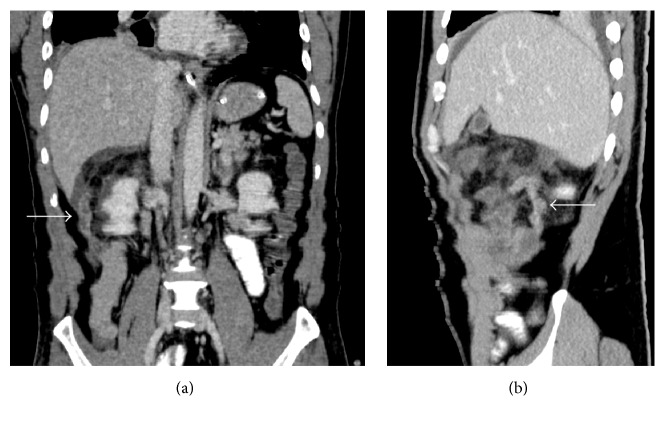
(a) Coronal image and (b) sagittal image of the contrasted CT scan showing the subhepatic appendix (white solid arrow).

**Figure 2:**
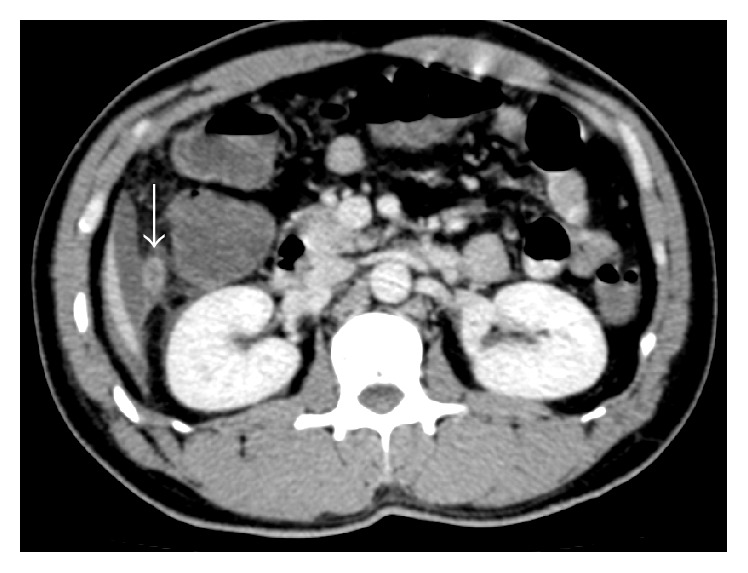
Transverse image of the contrasted CT scan showing the subhepatic appendix (white solid arrow).

Consequently, the CT scan revealed a retrocaecal thickened tubular structure at the right hypochondriac region, located subhepatically, extending cranially from the base, making a bend with its tip pointing at the epigastrium (Figures 1 and 2). We revised our diagnosis to acute appendicitis of subhepatically located appendix. With the consideration that the conventional Lanz incision may not provide the best access and the pathology was atypical, we proceeded with diagnostic laparoscopy for its versatility, aiming at removing the subhepatic appendix laparoscopically.

We performed the laparoscopy via a supraumbilical 10 mm optic port and two 5 mm working ports at both iliac fossae. During the procedure, we found that his ascending colon was short and that the caecum was located just below the liver at the right hypochondriac region. To reveal the retrocaecal appendix, we cut the lateral peritoneal reflection to medialize the caecum and ascending colon. The appendix was gangrenous, retrocaecal, and subhepatically located, in agreement with the CT scan findings. We attempted to separate the appendix from the caecum and ascending colon but were not successful due to technical difficulties and dense adhesion between the appendix and the caecum.

In consideration of the patient's safety, we carefully selected the best entry site laparoscopically and performed a transverse incision at the right upper flank near the hypochondriac region for its easy access to the subhepatic appendix. The open appendicectomy was uneventful. Postoperatively, the patient recovered well and has been discharged home on day 2 after the surgery. He remained well and was discharged from our clinic after 3 months of review.

## 3. Discussion

Subhepatically located caecum and appendix is a very rare phenomenon. It occurs due to the anomaly in fetal gut rotation that results in incomplete rotation and fixation of the intestine [3]. The earliest review of subhepatic caecum and appendix was published by King in 1955. In his review, such anomaly had been documented as early as 1863 [4]. To date, subhepatic appendix was only reported in 0.08% of all appendicitis, which equals an annual incidence rate of approximately 0.09 per 100,000 populations [5].

Due to the subhepatically located appendix, its inflammation mimics hepatobiliary or gastric pathology clinically [1, 2]. This may lead to delayed diagnosis and result in complications such as sepsis, suppuration, and perforation [5]. Therefore, radiologic imaging is of paramount importance in identifying such anomaly.

Ultrasound may be the preferred first-line screening tool due to its availability and ease to perform. However, there have been many reports where subhepatic appendiceal pathology was misdiagnosed as liver abscess or cholecystitis [2, 5]. On the other hand, although with radiation risk and less accessibility, computed tomography scan may provide high sensitivity (100%), specificity (95%), and accuracy (98%) in identifying acute appendicitis [6]. In our patient, the abdominal ultrasound only showed some free fluid in Morison's pouch, but the CT scan delineated the inflamed subhepatic appendix and excluded the other pathology.

The conventional Lanz incision in the right lower quadrant may not be suitable to remove the cranially situated subhepatic appendix. Therefore, laparoscopy is the optimal minimal invasive approach in this situation as compared with right hypochondriac region incision for its versatility, diagnostic, and therapeutic ability [7].

Laparoscopic appendicectomy can be difficult in retrocaecal subhepatic appendicitis where dense adhesion or fibrosis is present. To increase the success rate of such difficult laparoscopic appendicectomy, a few helpful technical tips are listed as follows [8]:

1. Use an angled telescope for better intraoperative view.
2. Mobilise the caecum early in the retrocaecal appendix for easier access.
3. Have an extra port for better access and traction.
4. You may twist the appendix during its traction to shorten it for easier dissection.

However, conversion to open approach may be unavoidable in difficult and complicated cases. The open approach has direct access to the pathology and is able to provide better tactile input. The decision to convert to open approach is for the patient's safety and should never be viewed as a failure.

## 4. Conclusion

Subhepatic appendicitis is a very rare but important differential diagnosis for the right upper acute abdomen. CT scan provides high accuracy in diagnosing acute appendicitis. Laparoscopy confers diagnostic as well as therapeutic value in managing this condition.
